# The Relevant Participation of Prolactin in the Genesis and Progression of Gynecological Cancers

**DOI:** 10.3389/fendo.2021.747810

**Published:** 2021-10-21

**Authors:** Adrián Ramírez-de-Arellano, Julio César Villegas-Pineda, Christian David Hernández-Silva, Ana Laura Pereira-Suárez

**Affiliations:** ^1^ Instituto de Investigación en Ciencias Biomédicas, Centro Universitario de Ciencias de la Salud, Universidad de Guadalajara, Guadalajara, Mexico; ^2^ Doctorado en Ciencias Biomédicas, Departamento de Fisiología, Centro Universitario de Ciencias de la Salud, Universidad de Guadalajara, Guadalajara, Mexico; ^3^ Departamento de Microbiología y Patología, Centro Universitario de Ciencias de la Salud, Universidad de Guadalajara, Guadalajara, Mexico

**Keywords:** prolactin, prolactin receptor, carcinogenesis, cancer progression, gynecological cancers

## Abstract

Prolactin (PRL) is a hormone produced by the pituitary gland and multiple non-pituitary sites, vital in several physiological processes such as lactation, pregnancy, cell growth, and differentiation. However, PRL is nowadays known to have a strong implication in oncogenic processes, making it essential to delve into the mechanisms governing these actions. PRL and its receptor (PRLR) activate a series of effects such as survival, cellular proliferation, migration, invasion, metastasis, and resistance to treatment, being highly relevant in developing certain types of cancer. Because women produce high levels of PRL, its influence in gynecological cancers is herein reviewed. It is interesting that, other than the 23 kDa PRL, whose mechanism of action is endocrine, other variants of PRL have been observed to be produced by tumoral tissue, acting in a paracrine/autocrine manner. Because many components, including PRL, surround the microenvironment, it is interesting to understand the hormone’s modulation in cancer cells. This work aims to review the most important findings regarding the PRL/PRLR axis in cervical, ovarian, and endometrial cancers and its molecular mechanisms to support carcinogenesis.

## Introduction

Prolactin (PRL), belonging to the PRL/growth hormone (GH)/placental lactogen family, is a polypeptide and pleiotropic hormone but mainly recognized as a lactogenic hormone and is primarily synthesized and secreted from the lactotroph cells of the anterior pituitary gland (1–5). Besides, PRL is also synthesized in multiple non-pituitary sites, including the endometrium, myometrium, decidua, immune cells, brain, breast, prostate, skin, and adipose tissue (2–6), acting as a paracrine/autocrine signaling molecule (5), and locally as a growth-promoting factor (2, 3, 7). PRL participates in various physiological events, performs its functions in the beginning and maintenance of implantation, pregnancy, and lactation, as well as proliferation and differentiation of mammary glands cells, immunoregulation, and angiogenesis (2, 4, 6).

In gynecological cancers, the interaction between PRL and the prolactin receptor (PRLR) mainly promotes protumoral events, such as migration, invasion, metastasis, chemoresistance, inhibition of apoptosis, among others, through the activation of different signaling pathways and effector proteins, which will be addressed later. Numerous reports show evidence of the effect of PRL-PRLR promoting cellular malignant characteristics in gynecological cancers, and there are even a few reports suggesting a possible anti-tumor effect generated by this ligand-receptor binding. In this review, we analyze these findings, and we present the current panorama of research on PRL-PRLR and its effects on gynecological cancers and the perspectives on the use of PRL as a therapeutic target.

## PRL as a Therapeutic Target, Pro- or Anti-Tumor?

Considering the existing evidence of the critical role played by PRL in gynecological neoplasms, some therapeutical strategies are being investigated, such as the blockade of PRL/PRLR signaling. Wen et al. utilized an antagonist peptide of PRL, G129R, which blocked the tumoral PRL/PRLR axis in orthotopic mouse models of human OCs, causing inhibition of tumor growth; they reported a synergistic effect of G129R with paclitaxel, which resulted in >90% lower tumor weights compared to controls (8). In a preclinical study, the activity of the high affinity anti-PRLR IgG1 antibody REGN2878-DM1 was evaluated, which induced a potent cell-cycle arrest and cytotoxicity in PRLR-expressing tumor cell lines. *In vivo* tests demonstrated significant antigen-specific anti-tumor activity against BC xenograft models and a synergistic effect when combined with fulvestrant (9).

Although there are numerous studies where the protumor effect of PRL is demonstrated, some reports do not support this idea or show the opposite. In a phase I clinical trial, the monoclonal antibody LFA102, a humanized neutralizing monoclonal antibody directed against the extracellular domain of PRLR, was tested in patients with advanced BC (10), which had previously caused tumor shrinkage in an *in vivo* model of BC (11). *In vitro* and *in vivo*, preclinical reports suggest that the PRL-PRLR union is vital for breast carcinogenesis (9, 12–21). However, in the clinical area, LFA102 had no anti-tumor effect when administered as a single agent in patients with PRLR-positive metastatic BC, questioning the role of PRL in the development of BC and highlighting the need for further studies to fully understand the role of PRLR-driven signaling cascades in tumor growth (10). Additionally, PRL has been shown to have antitumorigenic effects in BC cells that overexpress HER-2 by suppressing the epithelial-mesenchymal transition process; it also decreased the potential for malignancy by promoting cell adhesion and suppressing proliferation, tumor initiation/growth, drug resistance, tumorsphere formation capacity, and invasion of BC cells. Its expression was associated with favorable clinicopathological characteristics and better patient survival outcomes (22–25). This set of studies suggest PRL as a biomarker of good prognosis in BC and propose it as a therapeutic tool due to its demonstrated antitumorigenic role.

Supporting this order of ideas, López-Ozuna et al. showed that PRL induces the arrest of the cell cycle, modulates the expression of essential regulators of the cell cycle, induces heterochromatin formation, differentiation/senescence-associated morphological changes and resistance to growth signals, suppresses tumorigenesis *in vivo* resulting in the loss of proliferation and stemness in TNBC cells (26). The authors of this work suggest PRL as an adjuvant therapy to treat specific subtypes of TNBC, together with chemotherapy and other new targeted drugs such as cell cycle checkpoints inhibitors (26).

Full-length PRL (23 kDa), which has proangiogenic activity, can be proteolytically cleaved to produce a fragment of 16 kDa. This lower molecular weight isoform product of native PRL impairs functional tumor neovascularization by altering Notch signaling, particularly due to its inhibitory effect on vessel maturation through its binding to fibrinolytic inhibitor plasminogen activator inhibitor-1 (PAI-1). This is the reason why it is proposed as a promising anti-tumor candidate (27, 28). Due to the pro- and anti-tumor evidence of PRL existing in the literature, in this review we delve into this topic to provide an overview and understand the role of PRL in gynecological cancers.

## Effect of PRL on Mechanisms That Promote Gynecological Carcinogenesis

PRL promotes the initiation and progress of different gynecological neoplasms through different mechanisms. Furthermore, it is known that they share the cellular effects exerted through the JAK/STAT signaling cascade, such as survival, cell cycle progression, proliferation, migration, high metabolic rates, angiogenesis, and anti-apoptosis (9, 12, 29–36). In addition to this, it has been found that the PRLR is overexpressed in breast (14, 37), cervical (38–40), ovarian (41, 42), and endometrial cancer (EC) (2, 43) in comparison with their respective cancer-free tissues. Those mentioned above strongly propose PRL as a critical factor in promoting different mechanisms that favor carcinogenesis.

### PRL-Mediated Proliferation and Its Role in Cancer Cell Survival

Another mechanism important for PRL to support tumorigenesis is by inhibiting apoptosis of cancer cells. A study developed in ovarian cancer (OC) cells showed that apoptosis of cells expressing PRLR was diminished after pretreatment with PRL (44). Other research found that the suppression of apoptosis by PRL reflects its effects on the activation of multiple signaling pathways that lead to the regulation of survival proteins of the Bcl-2 family, particularly by enhancing the expression of Bcl-xL (45). Gadó et al. investigated the effect of PRL on the inhibition of apoptosis on multiple myeloma cells. They found that the effect exerted by PRL depended on the applied dose: higher doses inhibited apoptosis, whereas near-physiological doses exerted a pro-apoptotic effect (46). It has also been reported that PRL can increase the viability and decrease the apoptosis of human luminal-A BC cell lines by activating survival pathways involving enzymes such as metalloproteinase carboxypeptidase-D and EDD E3 ubiquitin ligase and the protein Ki-67 (18–21, 47).

Furthermore, it has been observed that treatment with PRL results in a protective effect conferring inhibition of apoptosis in cervical cancer (CC) cell lines (39). Ramírez de Arellano et al. also demonstrated in CC cell lines that treatment with PRL leads to the activation of STAT3, generating an increase in the level of anti-apoptotic proteins such as Bcl-xL, Bcl-2, and survivin (32). A microarray analysis performed with MCF7 BC cells showed that the binding of PRL with PRLR could induce the 1.2-fold upregulation of approximately 4,700 genes, which are involved in the activation of pathways related to neoplasm proliferation and progression (13).

By using a miRNA array in T47D human breast cancer (BC) cells, it was shown that PRL upregulates 21 miRNAs through MAPK/ERK and PI3K/Akt pathways. one of them, miRNA-106b, was strongly induced, and it promoted the expression of the mesenchymal markers, such as SNAIL-2, TWIST-2, vimentin, and fibronectin. It also favored cell migration and decreased the expression of the cell cycle inhibitor p21, contributing to tumor promotion (48). It has been shown that when immortalized cells are treated with PRL for a long time, it generates alterations in cell morphology, significantly increases the clonogenic capacity, and gives the cells the capacity to generate tumors, thus manifesting the promoter role of PRL in ovarian and endometrial tumorigenesis (49).

### PRL and Its Promoting Effect on Cancer Cell Migration

Migration is an essential cellular event that allows cancer cells to move towards new anatomical niches to generate secondary tumors, an action known as metastasis (12, 15). To achieve this, a reorganization of the actin cytoskeleton must be performed by the cell, and it is known that members of the RHO family of small GTPases, such as RAC, RHO, and CDC42 proteins, are essential controllers of cytoskeleton dynamics (50, 51). PRL actively participates in this event, maintaining a close relationship with multiple proteins; the binding of PRL to its receptor PRLR promotes motility, invasion, and metastasis of BC cells through modulation of c-Src, ezrin/radixin/moesin (ERM) family of actin-binding proteins, and FAK expression and phosphorylation. This modulation causes actin relocation toward the plasma membrane, which allows the formation of membrane ruffles and pseudopodia, where adhesion to extracellular proteins favors cell movement (16). Hammer and Diakonova demonstrated that PRL regulates BC cell lines’ migration through the pTyr-PAK1, MEK/ERK, and FAK pathways (17). This evidence highlights the important role that PRL has in cancer processes.

## PRL in Cervical Cancer

The first evidence indicating the role of PRL in cervical carcinogenesis came from the 1970 decade, where a study determined that PRL enhanced the proliferation of normal neonatal uterine cervix cultured cells from mice and in 3-methylcholanthrene-induced cervical carcinomas (52–54). A decade later, PRL was found to be present in normal, dysplastic, and malignant (adenocarcinoma and epidermoid carcinoma) human cervix. Strayer et al. identified that the expression of PRL was higher in the malignant and dysplastic cervix compared to normal tissue by immunohistochemistry (55).

In 1990, hyperprolactinemia was proposed as a biomarker of CC, mainly in the advanced stages of the disease (56). Later, in 1992, PRL was shown to increase the proliferation of the CC7-T and SiHa CC-derived cell lines (57), and elevated PRL levels were reported in the early stages of CC, and its level normalized once tumors were surgically removed, besides, it was demonstrated the ectopic PRL production by CC cells (58).

Regarding the expression of PRLRs, Dowsett et al. reported their absence in both normal and malignant uterine cervix (59). However, in 2013, a high expression of different PRLR isoforms (weighing 50, 60, and 110 kDa) and PRL variants (60-80 kDa) were reported in HeLa, SiHa, and C-33A CC cell line (39). PRLR was also increased in patients with low and high squamous intraepithelial lesions and CC, especially in this last group, by immunohistochemistry assays, concluding that high levels of PRLR in the CC patients correlated to the degree malignancy of the cervical tissues. Long and short isoforms were also identified in CC tissues by western blot and only short ones in cervical intraepithelial neoplasia samples. Besides, a 60 kDa PRL-like molecule was reported in cervical intraepithelial neoplasia and CC (38). Furthermore, recombinant PRL showed a protective role against apoptosis in CC-derived cell lines (39), where STAT3 was required to induce *Bcl-xl, Bcl-2, survivin*, and *Mcl-1* anti-apoptotic genes (32).

The 60 kDa PRL-like molecule was observed to be produced by CC cell lines and secreted to the extracellular medium, which allowed to carry out a magnetic bead hybridization protocol for its isolation and purification. The isolated 60 kDa PRL-like molecule was used to evaluate its effect on apoptosis in CC-derived cell lines, and it was found that apoptosis was inhibited when treated along with etoposide through the activation of the STAT3 pathway, suggesting a protective role of PRL to cell death by modulating the expression of Bcl-XL and survivin antiapoptotic proteins (40). Besides, PRLR was co-expressed with estrogen receptors α and β by an automated immunohistochemical study of serial sections, increasing along the different stages of cervical carcinogenesis. As well, the 60 kDa PRL-like molecule modified different cellular responses such as the following: increased the proliferation index of SiHa cell line, whereas in this cell line incubated with cisplatin in combination with E2 increased the apoptosis, and promoted metabolic activity in HeLa cell line, as well as in the non-tumorigenic keratinocytes HaCaT cell line transduced with HPV-18 E6 and HPV-16 E7 oncogenes. The interaction of E2 with the 60 kDa PRL-like molecule significantly affected metabolism but not cell survival. The combination of both hormones increased the metabolic activity of HeLa cells, measured by the MTT assay. However, their proliferation, evaluated by the xCELLigence platform, did not significantly change after the stimulus of both hormones. When analyzing the effect of E2 *versus* the combination with the 60 kDa PRL-like molecule on the MTT assays, HeLa and SiHa cells show that the 60 kDa PRL-like molecule seems to negatively modulate the estradiol´s effects on the metabolism of HeLa and SiHa cell lines (60).

The 60 kDa PRL-like molecule modulates the effects of E2 and the expression of its receptors in cell lines derived from cervical cancer (60); it would be interesting to know more about the regulation that the 60 kDa PRL-like molecule exerts on the expression of the genes induced by E2. An RNAseq analysis focused on cancer and metabolic pathways in cell lines positive for HPV infection would be helpful to confirm the role of the 60 kDa PRL-like molecule in this context.

While it is true that HPV infection is the main cause of CC, little has it been studied about the relationship between PRL and Human Papillomavirus (HPV). However, it was recently observed that treatment with PRL induces the expression of E6 and E7 oncogenes of HPV16/18 in HeLa and SiHa cell lines by a qRT-PCR assay. Besides, using HaCaT cells transduced with E6 and E7 oncogenes from HPV16 and HPV18, the authors reported that these oncogenes significantly increased the expression of PRLR (8.98–19.08 folds more than the control). Using the same cell lines, it was determined that both HPV18’s E6 and E7 oncogenes induced the relocation of this receptor from the cell membrane and from the cytoplasm to the nucleus, thus generating a loop that contributes to the development of CC, highlighting the existence of a PRL-HPV oncogenic regulation (61).

Even though there are several reports about the role of PRL impact in the CC, mainly by modulating vital processes such as proliferation, apoptosis, and mitochondrial activity, its role in migration and invasion is yet to be evaluated. The recent information proposes that PRL might interact with other hormones, like estradiol, and modify their activity in CC. Further research is required to establish this hormonal interplay.

## PRL and Ovarian Cancer

OC is the deadliest among gynecological cancers, partly because of the difficulty of its diagnosis. Because the ovaries are hidden in the peritoneal cavity, 75% of ovarian cancers are not diagnosed until they reach stage III or IV after metastasis has taken place (62). PRL has been proposed as a biomarker to diagnose OC because it is involved in many processes that support carcinogenesis.

Women with OC present high serum PRL (63); however, the question arises: Does this refer to a biomarker or a tumorigenesis factor? (42). Although this is an unanswered question, several findings point out the relevance of PRL in OC development, and several mechanisms have been proposed for PRL to promote tumorigenesis.

A study showed that after T29 ovarian epithelial cells were chronically exposed to PRL, carcinogenesis was induced, and this change resulted in dependence on the activation of Ras (49). PRL could induce carcinogenesis by regulating gene expression, for instance, *CD24^+^*, whose overexpression is associated with the tumor development and metastasis of human cancers (64, 65). This overexpression may have a meaningful impact on resistance to treatment. A study showed that ovarian carcinoma cells incubated with cisplatin reduced apoptosis when they were treated with PRL. These observations are hypothesized to be because PRL activates PI3K/Akt and induces the expression of anti-apoptotic genes, such as *Bcl-2*, which is seen in different types of cancers (44). Besides, CD24^+^ ovarian cells exhibit high resistance to antitumoral drugs, such as cisplatin and doxorubicin, and seem to be more susceptible to the lysis for NK cells (66).

The primary source of systemic PRL is hypophysis; however, several cells and tissues produce extra-pituitary PRL, which acts mainly in an autocrine and paracrine manner (67). Exogenous PRL has been an essential survival factor for OC by inhibiting apoptosis in these cells (44). The expression of PRL and its receptor in ovarian cancer cells suggests a possible PRL loop to support growth in an autocrine manner. This hypothesis is supported by Tan et al., who reported a decrease in viable cell numbers when incubating them with a PRLR antagonist (G129R-PRL) (41). Adding extra PRL did not increase the viable cell counts; however, when serum was removed, and cells became stressed, PRL increased up to 40% the cell number. This finding shows that exogen and autocrine PRL are important for ovarian cancer cells to grow.

PRL can also support ovarian tumorigenesis by activating signaling pathways associated with proliferation and inhibition of apoptosis. PRL can phosphorylate STAT5, mTOR, and ERK in OC cells leading to proliferation (42). The OVCAR3 ovarian cell line expresses activation of ERK1/2, MEK1, STAT3, and CREB after 30 minutes of a stimulus with PRL (49). Besides, positive feedback might be established because PRL can also activate Insulin-like growth factor (IGF)-1 and IGF-2, which induces PRL expression in a Ras-dependent fashion (68, 69). Interestingly, p53 has some residues (S15, S46, and S392) susceptible to phosphorylation by PRL. It has been proven that this phosphorylation stabilizes p53, leading to lower apoptosis and higher resistance against chemotherapeutic agents (70). The latter shows that PRL in OC is very important for tumorigenesis; thus, a panel for early detection of this pathology, called OVASURE, included PRL among the other five molecules (71). PRL has been retested recently, along with other proteins, and it is concluded to be an efficient diagnostic marker for OC with a *BRCA1* mutation (72).

Regarding *BRCA1*, PRL has been reported to inhibit its activity by interfering with p21 expression, a cell cycle inhibitor, in a STAT5-dependent manner in a BC cell-line model (73). Nevertheless, finding high levels of only PRL in the serum does not explicitly differentiate OC.

*In vitro* studies show that the PRL/PRLR axis is active in OC cell lines, promoting proliferation, cell migration, and survival (41). According to a microarray study, the PRLR was overexpressed in OC tumors, and over 98% expressed it (49, 74). The role of PRLR in OC seems to be essential for maintaining the tumoral status: PRLR deletion in OVCAR3 cells leads to blockage of tumor formation (42). The Ras protein can stabilize PRLR; thus, PRL-dependent activation of Ras in ovarian cells might provide a feedback to increase its actions (75).

Finally, some infections can also regulate the PRL/PRLR axis, thus regulating cancer promotion. Human cytomegalovirus (HCMV) increases the expression of PRLR and induces PRL at the transcriptional and protein level in OC cells (76). Because PRL acts as a growth factor, HCMV can induce ovarian tumorigenesis through this mechanism. Moreover, HCMV proteins are highly expressed in ovarian tumor tissues, and it has been associated with poor prognosis (77, 78). A possible direct and indirect effect might be performed by HCMV because most of the HCMV-cells in the tumor expressed PRLR. Even though PRL/PRLR axis is important during HCMV infection, the virus would use this pathway to induce cell proliferation; however, other pathways may also be modulated by the virus, such as EGFR, PDGFR, FGF, ETBR, and IGF (76).

PRL and PRLR have an essential role in OC, as documented in several reports. However, more research should be conducted regarding its molecular effects and mechanisms, such as signaling pathways inhibition or the 60-kDa PRL-like molecule assays, to clarify its actions and direct diagnostic and therapeutical strategies.

## PRL and Endometrial Cancer

The endometrium is one of the extra-pituitary anatomical sites where PRL is synthesized (2, 4, 6). PRL is vital in the correct physiology of this tissue, and it is a marker of the cellular process called decidualization, which consists of the differentiation of human endometrial stromal cells into specialized, epithelioid decidual cells that govern various aspects of embryo implantation and placenta formation, an integral step in the establishment of pregnancy (79–81). Upon transformation, decidualized cells acquire a secretory epithelioid-like phenotype, with PRL and insulin-like growth factor binding protein 1 (IGFBP1), the main markers of this differentiation process (66, 79, 80, 82–87). Several aspects regarding this hormone´s roles in the endometrium have been studied, such as its expression, the activation of cancer-associated signaling pathways, and its biological effect on decidualization, making PRL a relevant factor in the carcinogenesis and progression of EC.

Worldwide, among the gynecological cancers, EC is in the second position in incidence and 5-year prevalence and occupies the third position in mortality. The estimated number of new cases in 2020 was 417,367, and it was the cause of 97,370 deaths in the same year (88–90). The GLOBOCAN project, considering the incidence, mortality, prevalence, and age group for EC worldwide in 2020, estimates that by 2040 the incidence will increase 30.3%, which is equivalent to 126,343 new cases for a total of 543,710, and even more worryingly, it is estimated that mortality will increase 49.8%, which means that in the next 20 years, there will be 48,473 deaths as a consequence of this neoplasm (90–92).

There is evidence that PRL promotes carcinogenesis and progression from the early stages of EC (4, 43, 93, 94). Ding et al. demonstrated that autocrine PRL expression could stimulate cell proliferation, anchorage-independent growth, migration, invasion of EC cells and tumor growth, local invasion, and metastatic colonization in xenograft models. They also reported that forced expression of PRL decreased the sensitivity of EC cells to chemotherapeutic drugs (i.e., doxorubicin and paclitaxel), both *in vitro* and *in vivo* (2). Therefore, they propose that inhibiting the signaling generated by PRL is a potential therapeutic target in late-stage EC (2). Furthermore, it has also been shown that PRL expression, either individually or in combination with GH expression, correlates with the stage of the disease, myometrial invasion, and is associated with worse relapse-free survival and overall survival in patients with EC (95).

PRL-induced cell proliferation occurs in endometrial carcinoma cells due to its binding with PRLR, which generates phosphorylation of ERK1/2, MEK-1, STAT3, CREB, ATF-2, and p53, and activation of various transcription factors (7, 49). Moreover, chronic exposure of human immortalized normal ovarian epithelial cells to PRL causes their malignant transformation and confers them the ability to form tumors in SCID beige mice (49). The PRLR’s expression is increased in endometrial hyperplasia and tumors, showing a vital role of the PRL/PRLR loop in EC. Something to note is that the expression of PRLR depends on several factors, one of them is the presence of proinflammatory cytokines, such as TNF-α, IL-1β, and IFN-γ, so those diseases and conditions that generate a chronic inflammation can also be supporting EC through the induction of PRLR. A report showed that the expression of PRLR was negatively correlated to the grade of EC, meaning that PRLR’s expression is higher in the initial stages and almost null by the late ones; which led the authors to think that PRL is more critical for the early neoplastic transformation than in developing the tumor (49).

In general, the evidence associates PRL with EC progression; however, although few, there are controversial reports that have proposed it as a suppressor of this neoplasia (96, 97). Imai et al. proposed that the protein kinase C in the endometrial fibroblasts must be stimulated by PRLR occupancy and that chronic stimulation by PRL may induce downregulation of protein kinase C in the fibroblast with a consequent decline of mitogenic activity, concluding that PRL could suppress mitogenesis (96). Another controversial report, carried out in rats, proposes that PRL may have an inhibitory effect on uterine carcinogenesis (97).

Classically, a biomarker is defined as an antigen or protein expressed by the tumor itself or the tumor microenvironment in response to the tumor. Thus, biomarkers are cellular or soluble indicators of the physiological state during the development of a disease. The best biomarkers have high sensitivity, high specificity, and can be evaluated in samples that do not require invasive procedures, such as it is serum and other biological samples (98). Serum PRL levels are considerably elevated in patients with EC compared to levels in cancer-free patients (4, 49, 93, 94, 99), as well as PRLR, and PRL mRNA are overexpressed in endometrial tumors (4, 49). Erdenebaatar et al. proposed that elevated serum PRL levels may contribute to the tumorigenesis of organs that express the PRLR (93). Considering the increased levels of PRL in the serum of patients with EC, compared to the serum PRL levels of patients with other types of neoplasms, PRL has been proposed as a strong, sensitive (98.3%) and specific (98.0%) biomarker for the early detection of EC (100), and its combination with other biomarkers could improve the sensitivity and specificity of the EC diagnosis (2, 4, 6, 100–102).

Signaling pathways activation has also been reported to be associated with the development of endometrial malignancies. Ras signaling has also been implied in EC development, and some authors postulate PRL to activate Ras oncogenes potently. The activation of Ras leads to PRLR stabilization (75). PRL induces the expression of IGF-1 and -2, and their signaling leads to the expression of PRL in a Ras-dependent way (68, 69). The PRL/PRLR loop’s activation can originate proliferation, apoptosis resistance, angiogenesis (103), activate signaling pathways related to cell adhesion and motility, and promote endometrial tumorigenesis (104).

Since PRL is recognized as a biomarker (93, 100), poor prognosis marker (95), risk factor (49, 105–107), and has even been proposed as a potential therapeutic target (2, 43) for EC, it would be fascinating to carry out clinical studies in which the interaction of PRL with PRLR is blocked to confirm the evidence shown by the various working groups that propose PRL as a critical factor in the progression of this type of neoplasia.

## Conclusions

**Figure 1:**
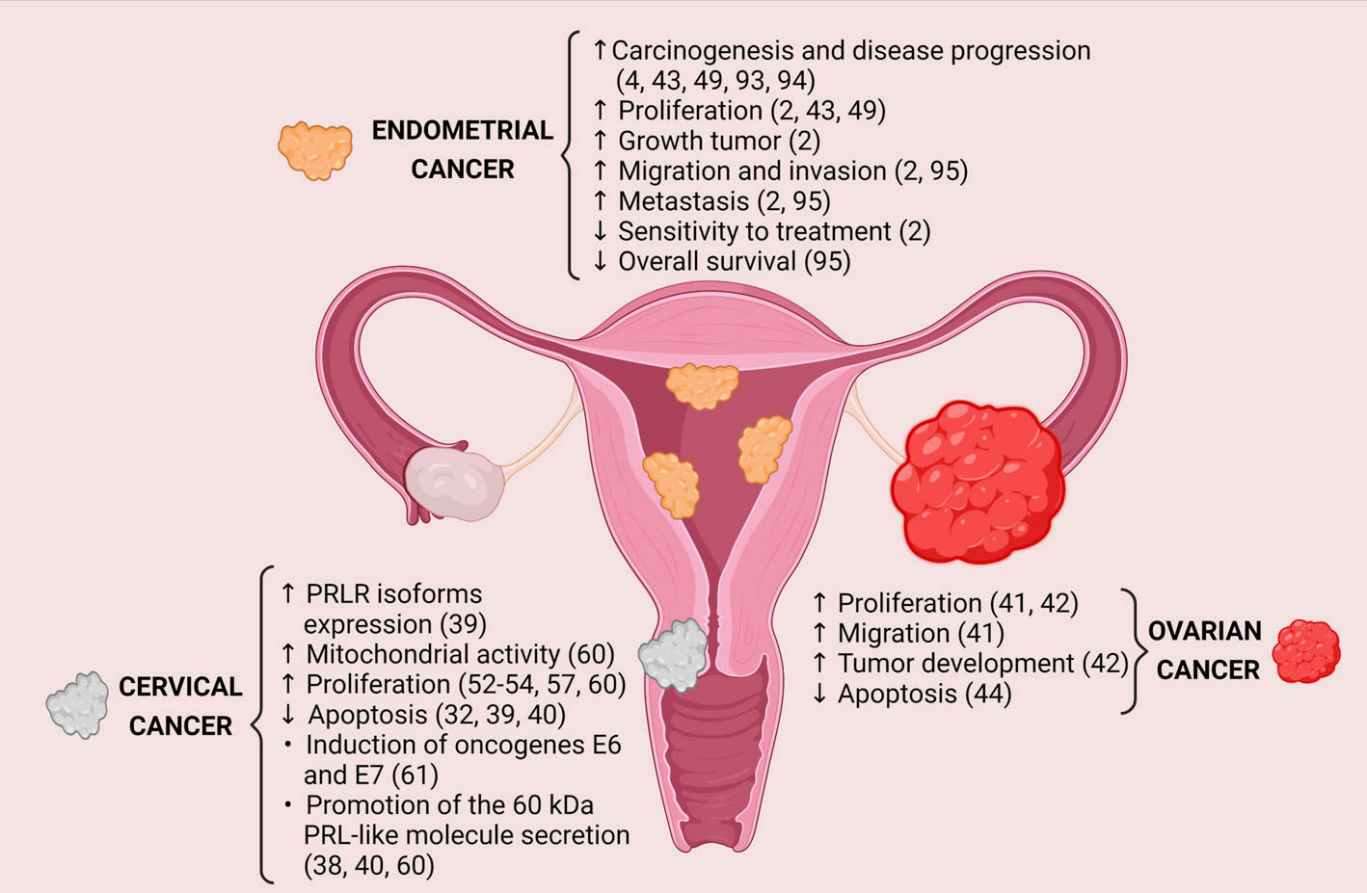
Protumoral effects generated by PRL-PRLR interaction in gynecological cancers. Image created in BioRender.com.

PRL exerts different biological effects that promote carcinogenesis and the progression of gynecological tumors, and its expression may induce several processes as survival, cellular proliferation, migration, invasion, metastasis, and resistance to treatment (**Figure 1**). The PRLR has been found widely expressed in the cervix, ovary, and endometrium cancers compared to their healthy tissues. However, little is known regarding the modulation of the signaling pathways activated by the PRLR and their target genes´ expression. Besides, it has been observed that some infections can regulate the PRL/PRLR axis, thus favoring cancer progression (**Table 1**). For example, the relationship between PRL and HPV; PRL induces the expression of E6 and E7 oncogenes of HPV16/18 in SiHa and HeLa cell lines, respectively, and HPV oncogenes increase the levels of PRLR.

**Table 1:** Relevance of PRL in gynecological cancers.

Model	Relevance in cancer	References
**CERVICAL CANCER**		
-Mice: uterine cervix cells and CC induced with 3-methylcholanthrene	-PRL increased cellular proliferation	Forsberg et al. (52), Forsberg et al. (53), Forsberg et al. (54)
-Patients: Biopsies	-High PRL expression in the malignant and dysplastic vs normal tissues	Macfee et al. (55)
-CC Patients: Serum	-Hyperprolactinemia was proposed as a biomarker in the advanced stages of cancer	Strukov et al. (56)
-CC cell lines: CC7-T and SiHa	-PRL increased cellular proliferation	Chen et al. (57)
-CC Patients: Serum	-High PRL levels in early stages of CC	Hsu et al. (58)
-CC cells explant	-PRL levels normalized in tumor surgically remoting ectopic PRL production	
-CC Patients: Biopsies	-Absence of PRLRs	Dowsett et al. (59)
-CC cell lines: HeLa, SiHa and C-33A	-High expression PRLR isoforms (50, 60, and 110 kDa) and PRL variants (60-80 kDa)	Lopez-Pulido et al. (39)
	-rPRL showed a protective role against apoptosis	
-CC cell lines: HeLa, SiHa and C-33A	-High levels of PRLR in the CC patients	Ascencio-Cedillo et al. (38)
	-PRLR isoforms: (Long and short in CC, short in CIN and normal tissues)	
	-The 60 kDa PRL-like molecule was found in CIN and CC	
-CC cell lines: HeLa, SiHa and C-33A	-rPRL and the 60 kDa PRL-like molecule induce *Bcl-xl, Bcl-2, survivin*, and *Mcl-1* genes *via* STAT3	Ramírez De Arellano et al. (32)
	-The 60 kDa PRL-like molecule inhibited apoptosis	Ramírez De Arellano et al. (40)
-CC Patients: Biopsies, CIN and CC	-PRLR, ER-α and -β co-expressed in CC.	Riera-Leal et al. (60)
-CC cell lines: HeLa and SiHa	-PRL increased proliferation and apoptosis of SiHa cell line	Ramírez-López et al. (61)
-HaCaT cell line transduced with HPV-18 E6 and HPV-16 E7 oncogenes	-Promoted mitochondrial activity	
	-PRL induces the expression of E6 and E7 oncogenes of HPV16/18 –	
	-E6 and E7 HPV oncogenes increased PRLR expression in nuclei	
**OVARIAN CANCER**		
-OC Patients: serum	-Higher PRL levels have been proposed as a biomarker	Mor et al. (63)
-OC cell line: T29	-Appearance of OC after chronic exposition to PRL	Levina et al. (49)
-OC cell lines: OVKATE and OVISE	-PRL activates PI3K/Akt and induces anti-apoptotic genes expression, e.g. *Bcl-2*	Asai-Sato et al. (44)
-OC cell lines: TOV21G, OV90, and TOV112D	-Decreased cell viability when treated with a PRLR antagonist	Tan et al. (41)
-OC cell line: OVCAR3 and human FT33-Tag-Myc cells	-PRL can phosphorylate STAT5, mTOR, and ERK in OC cells leading to proliferation	Karthikeyan et al. (42)
	-PRLR deletion in OVCAR3 cells leads to blockage of tumor formation	
**ENDOMETRIAL CANCER**		
-EC Patients: serum	-Serum biomarker, PRL increased in EC patients	Nithin et al. (101)
-EC Patients: serum	-Serum biomarker, PRL was the strongest biomarker to discriminate EC from other gynecological cancers, providing 98.3% sensitivity and 98.0% specificity	Yurkovetsky et al. (100)
-EC Patients: biopsies	-PRL expression was associated with FIGO stage, myometrial invasion, and with worse relapse-free survival and overall survival in EC patients	Wu et al. (95
-EC cell lines: Ishikawa and RL95-2	-PRL signaling inhibition is a potential therapeutic strategy for late-stage EC treatment	Ding et al. (2)
	-PRL stimulated proliferation, anchorage-independent growth, migration, invasion, and decreased sensitivity of EC cells to chemotherapeutic drugs	
-EC cell line: Ishikawa	-PRL enhanced the proliferation of EC cells	Yamaguchi et al. (43)
-EC cell lines: HEC-1A, AN3 CA, and RL95-2	-PRL may represent a risk factor for EC	Levina et al. (49)
	-PRL potently induced cell proliferation	
	-PRL activated Ras oncogene	

PRL, prolactin; rPRL, recombinant prolactin; PRLR, prolactin receptor; ER, estrogen receptor; CIN, cervical intraepithelial neoplasia; CC, cervical cancer; OC, ovarian cancer; EC, endometrial cancer; HPV, human papillomavirus; FIGO, International Federation of Gynecology and Obstetrics.

Nevertheless, it would be essential to elucidate the molecular mechanisms of the PRL/PRLR axis involved in cervical carcinogenesis. Furthermore, PRL is recognized as a biomarker of endometrial cancer development and poor prognosis, thereby it has been proposed as a potential therapeutic target, but clinical studies need to be carried out to understand its modulation. The role of PRL in cervical and ovarian cancer has been less investigated; however, the evidence suggests that this hormone could be strongly involved in promoting carcinogenesis through different mechanisms.

## Author Contributions

AP-S conceptualized and drafted the manuscript. JR-d-A, JV-P, CH-S, and AP-S participated in the bibliographic research, writing, design of the figure, and review of the article. All authors contributed to the article and approved the submitted version.

## Funding

This work was supported by Sectorial Research Fund for Education, SEP-CONACYT (A1-S-51207), and Jalisco Scientific Development Fund (FODECIJAL) to Attend State Problems 2019 (Project #8168). JV-P received a CONACYT fellowship (#769371, 2nd Year of Continuity of Post-Doctoral Stays Linked to Strengthening the Quality of the National Postgraduate 2020(2), CVU: 377666).

## Conflict of Interest

The authors declare that the research was conducted in the absence of any commercial or financial relationships that could be construed as a potential conflict of interest.

## Publisher’s Note

All claims expressed in this article are solely those of the authors and do not necessarily represent those of their affiliated organizations, or those of the publisher, the editors and the reviewers. Any product that may be evaluated in this article, or claim that may be made by its manufacturer, is not guaranteed or endorsed by the publisher.
